# Ambra Grisea

**Published:** 1888-03

**Authors:** Horace Still


### AMBRA GRISEA.

(Read before Hahnemannian Association of Pennsylvania.)

I have selected this remedy for my paper, not because I expect to produce anything new, but because I have been recently interested in its study, and hope by enumerating what seems to me to be some of the more characteristic symptoms to provoke a discussion which may be beneficial to the members of the Association.

This substance, according to Hahnemann, is developed in the intestines of the sperm whale, and is probably a fatty secretion from its gall-bladder. It was first introduced by Hahnemann in 1827, and was especially proved by his friend, Count de Gersdorf, from whose provings we get many important symptoms.

### SYMPTOMS.

MIND.—Impaired memory; want of comprehension; being obliged to read everything over several times before it can be understood; difficulty of thinking in the morning; in impaired memory, *Anacardium Orientale* compares very strongly, it (Anacard.) having impaired memory worse in the morning, better in the afternoon, but his recollections only come to him after he is in need of them: the Anacardium patient, however, according to Hering, easily remembers what he reads, while the Ambra patient, on the contrary, has to read everything over several times in order to comprehend it. (A number of other remedies have impaired memory, among which may be mentioned Alumina, Aurum, Carbo veg., Lycop., Rhus, Verat., Zinc.)

Melancholy, with depression and loathing of life (Aurum); great weakness, pain in small of back, and constipation; sleeplessness after business embarrassments (also Act. r.); the presence of other people aggravates the symptoms.

SENSORIUM.—Vertigo, with feeling of weight on vertex; has to lie down on account of vertigo and feeling of weight in stomach.

HEAD.—Congestion to head from music; very painful tearing on top of the head and as if in the whole upper half of the brain, with pale face and coldness of the hands; tearing pain in the left temple up to top of head, on the right frontal protuberance, and *behind the left ear.*

OUTER HEAD.—On the right side of head, a spot where the hair when touched pains as if sore; falling off of the hair.

[COMPARISONS—ALOES. Hair comes out in lumps, leaving bare patches; eye-lashes also fall out; frequent frontal headache.

AMMON. MUR.—Large accumulation of bran-like scales, with falling of hair, which has a deadened appearance, with great itching of scalp.

ARSENICUM ALB.—Bald patches at or near forehead; scalp covered with dry scabs and scales.

CARBO VEG.—Falling off of hair after severe illness or confinement.

FLUORIC ACID.—Itching of head; falling off of hair; the new hair is dry, and breaks off.

KALI CARB.—Dry hair; rapidly falling off, with much dandruff.

PHOSPHORUS.—Bald spots behind the ears.

PHOS. AC.—Gnawing grief changes the hair of the young to gray.

VINCA MINOR—Hair falls out in single spots and white hair grows there; spots on head oozing moisture; the hair matting together.]

EYES.—Pressure in eyes as if they lay deep; eyes difficult to open in the morning; feeling as if they had been closed too firmly (A. M., also Nicc. and Nit. ac.; in the evening, *Cocculus* and *Sepia.*)

EARS.—Deafness of one ear—crackling in left ear.

NOSE.—Dried blood gathers in nose (nose continuously full of coagulated blood, *Ferrum*). Epistaxis early in the morning.

FACE.—Flushes of heat; jaundiced color of face; spasmodic twitching of face in the evening in bed (A. M. in bed, *Nux vom.*).

TEETH AND GUMS.—Drawing toothache at one time in right and at another time in left teeth, by day and at night; worse from warmth, momentarily better from cold; not aggravated by chewing and passing off after eating; bleeding of the gums; profuse bleeding out of the right lower teeth.

MOUTH.—Ranula; fœtor of mouth; blisters in mouth; pain as if burnt; under the tongue, lumps like small growths, which pain like excoriation; smarting and soreness of interior of mouth; on account of pain she could not eat anything hard. (Rasping, smarting pain in mouth when eating solid food, *Phos. ac.*) In the morning on awaking, tongue, mouth, and lips as if numb and dry (sensation of numbness in throat, *Mag.*, *Sulph.*) Tongue coated grayish yellow.

PALATE AND THROAT.—Sensation of rawness in velum pendulum palati; scraping in throat; accumulation of grayish phlegm in throat which is difficult to hawk up, accompanied by rawness. When hawking up mucus in the morning, almost unavoidable retching and vomiting; smarting at the back of the fauces when not swallowing.

STOMACH AND ABDOMEN.—Frequent empty or *sour* eructations; when walking in the open air heart-burn with baulked eructations; heart-burn from milk especially warm milk; after eating cough and gaping, and a feeling as if food did not go down into the stomach (food descends slowly and gets into the larynx, *Kali carb.*; food is felt until it enters the stomach, *Alumina*, *Bryonia*, *Phosphorus*).

Aching pain in a small spot in hepatic region, not felt when touched; coldness in abdomen, especially left lateral coldness; pain in the region of the spleen, as if something were torn off, tension and distention in abdomen after every mouthful he eats, even after every mouthful of fluid.

STOOL.—Constipation, frequent ineffectual desire; this makes him very anxious and the near presence of others is unbearable (near presence of others unbearable while urinating, *Nat. m.*); after stool, aching deep in hypogastrium; itching and smarting in anus relieved by rubbing.

URINARY ORGANS.—Urine turbid even when first passed; urine sour-smelling, frequent urination at night (Lyc.); burning, smarting, itching in vulva and urethra when urinating.

SEXUAL ORGANS.—*Male*—Voluptuous itching on the scrotum (*Staph.*); rawness between thighs. *Female*—Severe itching of pudendum, must rub the parts; discharge of bluish white mucus from vagina, only at night; preceding each discharge a stitch in the vagina; discharge of blood between the menstrual periods at every little accident; stitches in ovarian region when drawing-in the abdomen or pressing upon it; menses too early and profuse (*Aloes*, *Calc. c.*, *Rhus*, etc.; too early and scanty, *Alum.*, *Silic.*, etc.); constipation with tenesmus in child-bed; abdomen puffed, causing anxiety, and anxiety from the presence of others.

COUGH.—Spasmodic, from tickling in larnyx; spasmodic, with frequent eructations and hoarseness; cough, with pain under left ribs as if something were torn loose; night cough from excessive irritation in throat; expectoration yellow or *grayish*, tough, tasting salt or sour, only in the morning.

CHEST.—Asthma of old people and children; oppression in chest or between scapulæ; rawness in chest; itching or tickling in chest, causing cough (itching behind sternum causes violent, racking, paroxysmal cough, *Kali bichro.*); pain in lower part of right chest, relieved by lying upon it.

HEART.—Violent palpitation; pressure in chest as from a lump or as if the chest were stuffed full; anxiety at heart hindering respiration, with flying heat; pulse quick, feels pulse in body (*Nat. c.*).

EXTREMITIES.—Cramps in hands on grasping; arms "go to sleep" while lying upon them and at other times; tearing in left shoulder joint; twitching of limbs during sleep; limbs "go to sleep" easily; cramps in calves at night (*Sulph.*); tearing pain in left leg; heaviness of lower limbs; sense of contraction in right thigh, limb feels shorter (left limb seems shorter, *Causticum*); left leg distended and blue during menses; itching in interior of soles, not relieved by scratching.

SLEEP.—Coldness of body during sleep; twitching of limbs during sleep. This coldness and twitching often prevents sleep.

CHILL, FEVER, SWEAT.—Chill of single parts; chill with lassitude and sleepiness, relieved by eating (sweat lessened by eating, *Anacard.*); skin of whole body, except face, neck, and genitals, cold (ice-cold genitals, *Sulph.*).

HEAT.—Frequent flushes, worse in evening; sweat profuse every morning; worse on affected side (also *Ant. t.*); profuse sweat from least exertion, especially of abdomen and thighs.

AMBRA is adapted to all ill-natured, nervous, hysterical, lean, and aged persons; to one-sided complaints. It is said to reproduce the itch eruption.

AGGRAVATIONS.—In the evening; while sleeping; on awaking; from the presence of others.

AMELIORATIONS.—From lying on affected and painful side (Bry.).

RELATIONSHIP.—*Anacard.*, *Ars.* (asthma), *Act. r.* (night cough, business embarrassments), *Ignat.*, *Asaf.*, *Lyc.*, *Puls.*, *Lep.*, *Sulph.*, *Verat.* (cough with eructations).

It antidotes *Staph.*, especially the voluptuous itching of scrotum.

SOME VERIFICATIONS.—Aggravation of symptoms from presence of others; eyes difficult to open in the morning, feeling as if they had been closed too firmly; scraping in throat; accumulation of grayish phlegm difficult to hawk up, accompanied by rawness and almost unavoidable retching and vomiting; pain in the region of the spleen as if something were torn off, with a spasmodic cough, with eructations and hoarseness; expectoration tough, grayish mucus, usually worse in the morning; itching behind the sternum, causing cough; tearing in left shoulder joint; twitching of limbs, preventing sleep.

HORACE STILL, M. D.

